# Isolation of circulating tumor cells in non-small-cell-lung-cancer patients using a multi-flow microfluidic channel

**DOI:** 10.1038/s41378-019-0045-6

**Published:** 2019-02-25

**Authors:** Jian Zhou, Arutha Kulasinghe, Amanda Bogseth, Ken O’Byrne, Chamindie Punyadeera, Ian Papautsky

**Affiliations:** 1 Department of Bioengineering, University of Illinois at Chicago, Chicago, IL 60607 USA; 2 University of Illinois Cancer Center, Chicago, IL 60612 USA; 3 The School of Biomedical Sciences, Institute of Health and Biomedical Innovation, Queensland University of Technology, Kelvin Grove, QLD Australia; 4 Translational Research Institute, Brisbane, QLD Australia; 5 Princess Alexandra Hospital, Woolloongabba, QLD Australia

**Keywords:** Chemistry, Other nanotechnology

## Abstract

Circulating tumor cells (CTCs) carry a wealth of information on primary and metastatic tumors critical for precise cancer detection, monitoring, and treatment. Numerous microfluidic platforms have been developed in the past few years to capture these rare cells in patient bloodstream for deciphering the critical information needed. However, the practical need for a high-quality method of CTC isolation remains to be met. Herein, we demonstrate a novel multi-flow microfluidic device that is able to sensitively provide high purity (>87%) of separation outcome without labeling. Our device is constructed and configured based on the phenomenal effect of size-dependent inertial migration. The recovery rate of >93% has been achieved using spiked cancer cells at clinically relevant concentrations (10 cells per 5 mL and above). We have also successfully detected CTCs from 6 out of 8 non-small-cell-lung-cancer (NSCLC) patients, while none for 5 healthy control subjects. With these results, we envision our approach is a promising alternative for reliable CTC capture, and thus for facilitating the progress of extracting information from CTCs to personalize treatment strategies for solid tumor patients.

## Microfluidics: New system to isolate circulating tumor cells

A novel multi-flow microfluidic system has been devised to isolate circulating tumor cells (CTCs) in patients with non-small cell lung cancer (NSCLC). NSCLC accounts for around 85% of all lung cancers. CTCs are cells that have been released from a primary tumor and are transported around the body by the blood. Those cells carry information that is critical for precise cancer detection, monitoring, and treatment; however, a high-quality method for isolating CTCs has hitherto been lacking. A team headed by Ian Papautsky at the University of Illinois at Chicago developed a novel multi-flow microfluidic device that affords high fidelity in separating CTCs from the blood of NSCLC patients. The authors believe that their versatile device offers considerable potential for facilitating extraction of information from CTCs in NSCLC and other forms of cancer.

## Introduction

Isolation of viable and intact circulating tumor cells (CTCs) is critical for implementing liquid biopsy which has shown their strong clinical implications as an alternative to tissue biopsy^1,2^. These cells serve as promising biomarkers for cancer prognostics, monitoring treatment response, drug screening, and personalized medicine^3–5^. Liquid biopsy continuously receives emphasis due to a number of advantages over traditional tissue biopsy, including readily accessibility and the potential for dynamic monitoring of cancer progression^6,7^. It is unrealistic to implement conventional invasive biopsy frequently and for some cancers, such as brain cancer and lung cancer, they are extremely difficult to biopsy due to the tumor location^6,8^. CTCs, on the other hand, can be easily accessed since they are tumor cells shed from primary and/or distant sites and circulate in the bloodstream^9^. These cells carry a wealth of information about specific mutations the tumor tissue may possess, allowing for targeted therapy approaches. Mechanisms of treatment resistance can also be investigated through the analysis of CTCs^10^. However, isolation of CTCs from blood is nontrivial, chiefly attributed to their extreme rarity as compared to surrounding blood cells^9,11^.

Many technologies have been developed in the past decade to tackle the challenge of capturing CTCs. Although flow cytometry is capable of CTC isolation, its throughput is too low to be practical^12^. CellSearch^TM^ system (Menarini Silicon Biosystems, Italy) is the only FDA-approved commercial product for CTC enumeration^2^. Nevertheless, its wide adoption in clinical settings is hindered by its shortcomings such as high cost, manual process, and false-positive/false-negative^13^. Recently, the burgeoning development of microfluidic technology has jump-started the search of alternative solutions for a greater CTC isolation. Numerous microfluidic platforms have been proposed to address the challenge. These systems can be roughly classified into two groups^14^. One major group is affinity-based methods that utilize surface markers, such as EpCAM, to distinguish CTCs from their surrounding blood cells^2,15,16^. While these methods show very good specificity, they are hindered from false-negatives due to downregulation of the expression of surface markers on some CTCs which are undergoing epithelial–mesenchymal-transition (EMT)^2^.

The other group differentiates CTCs from blood cells based on the physical properties of CTCs, mainly the size and deformability differences of CTCs and white blood cells (WBCs)^2,14^. Due to the independence of surface markers, technologies of this group are deemed as critical complementary methods to affinity-based approaches for improved CTC isolation. These size-based, and thus label-free methods include acoustic and electrophoresis platforms^17,18^, hydrodynamic and cross-flow filtration^19,20^, micropore and micropost trapping^21–23^, deterministic lateral displacement (DLD)^24,25^, and inertial focusing systems^26–29^ (including viscoelasticity aided method^30^). Our recent work has also successfully demonstrated the separation of CTCs directly from patient whole blood based on cell size^31^. While many of these emerging systems have been tested using patient samples, they share a common shortcoming: their purity remains to be significantly improved^15^. High purity is in strong demand for CTC enumeration, molecular characterization, and functional assays with less background intervention from WBCs^9^.

In this work, we report on a novel multi-flow microfluidic (MFM) system for the separation of CTCs with high purity. The microchannel takes advantage of inertial migration of cells^32^. The lateral migration of cells strongly depends on cell size in our microchannel, and label-free separation of CTCs from WBCs is thus achieved without sophisticated sample preparation steps and external controls required by affinity-based and active approaches. We conducted a thorough investigation on the effect of flow rate and flow rate ratio of sample and buffer flows on the channel performance prior to the tests of using clinical samples from non-small-cell-lung-cancer (NSCLC) patients. Our results with spiked samples show superb separation efficiency (>99%) and purity (>87%). We recovered >93% of spiked cancer cells for a concentration of 50 cells or above per 5 mL diluted blood and >83% for a concentration of 10 cells per 5 mL diluted blood. Samples of 8 NSCLC patients and of 5 healthy donors were processed through our microchannel. We isolated CTCs from samples of 6 patients with a maximum of 78 CTCs per mL blood and no CTCs were found in healthy control samples. We demonstrate a simple protocol as a promising alternative to existing methods for CTC isolation with high fidelity.

## Results and discussion

### Design and working principle

Our separation microsystem is built upon the inertial migration of cells flowing in microchannels. From our earlier work^32^, cells are subjected to inertial forces that drive them toward equilibrium positions in two stages. First, cells migrate rapidly toward sidewalls of the microchannel undergoing shear-induced lift force. Then, in the second stage, cells slowly migrate to their equilibrium positions centered on each sidewall under the influence of the rotation-induced lift force (*F*_Ω_). In a rectangular cross-section microchannel, the equilibrium positions reduce to only two, centered the long faces of the cross-section. The lateral migration of cells in such a system is strongly dependent on cell size, with larger cells exhibiting faster migration. This size-based migration gives rise to the orderly spatial arrangement of cells along channel long face (width in a low aspect ratio channel) for a certain observation window^33^. In this case, cells position laterally according to their size: the larger the cell, the closer it is to the channel centerline. Such self-ordering offers a unique opportunity for size-based separation in a simple straight channel, with a premise of cells initially near channel sidewalls.

**Fig. 1:**
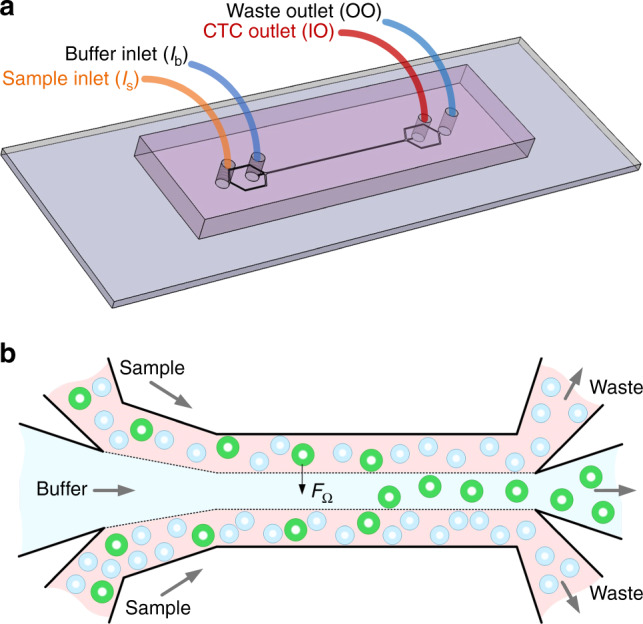
Device layout and schematic illustrating the working principle of inertial migration. **a** Configuration of the inlets and outlets. Blood sample and PBS buffer are introduced into the channel from sample inlet (I_s_) and buffer inlet (I_b_), forming the sandwiched flow configuration in the main channel as shown in (**b**). CTCs are separated and collected from the inner outlet (IO) and other blood cells smaller than the cutoff size are collected from the outer outlet (OO). **b** Topview of the multi-flow configuration enabling the size-dependent differential migration of cells toward buffer flow in the middle of the main channel. One of the main inertial forces, rotation-induced lift force (*F*_Ω_), is the predominant force behind the size-based lateral migration and subsequent isolation of target circulating tumor cells (CTCs) in this device

Our microchannel is designed to accommodate multiple flows to initially confine cells near channel sidewalls (Fig. 1). Briefly, the sample is introduced into the channel from the sample inlet (I_s_) and buffer solution is injected into the buffer inlet (I_b_). Through such configuration, three flow streams are formed in the main channel, with clean buffer flow in the middle to collect larger target cells which are subjected to size-dependent inertial forces of which rotation-induced lift force (*F*_Ω_) is predominant. By adding trifurcation, cells are subsequently separated into two groups based on their size (*a*).

Since our design is aimed at isolation of larger CTCs from the peripheral blood sample of NSCLC patients^34,35^, a size threshold must be set for the channel to appropriately extract target cells. Although our recent work has demonstrated the possibility of such flow configuration for CTC isolation directly from whole blood^31,36^, the considerable contamination from red blood cells (RBCs) compromises its outcome. Thus, in this work RBCs were first removed by a cell lysis protocol. As a result, we set the threshold size to be ~14 µm to differentiate CTCs from WBCs, which are mostly sized around 10 µm. We fixed our channel cross-section as 150 µm × 50 µm and thus the threshold size, also termed cutoff size, is largely determined by channel length. This length can be estimated from our earlier work^32^ with 20 µm particles in 100 µm × 50 µm microchannels. Considering that focusing length (*L*) varies inversely with the square of particle size (*L* ∝ *a*^−2^) for channels with similar characteristic lengths^37^, the focusing length was calculated as 17 mm for 14 µm cells. We set the length to be 20 mm to accommodate the influence of channel aspect ratio and the potential effect of flow rate ratio which largely defines initial positions of cells.

### Optimization of flow conditions

In our multi-flow microchannel, flow rate ratio can significantly influence separation performance. Due to the laminar nature of flows inside microchannels, cells in the sample flow near sidewalls would not cross the interfaces of sample and buffer flows (Fig. 1b) if no inertial effect takes place. Inertial forces drive cells laterally into the buffer flow in channel center. The velocity of such cross-flow migration is strongly dependent on cell size^32^. Ideally, we want larger target cells to achieve full focusing in the buffer flow while non-targeted cells are retained in sample side flows. In this case, collecting only the buffer flow at the end of the channel will yield a fraction of pure target cells. For our MFM microchannel with a given outlet system, fractionating the buffer flow is determined by the flow rate ratio which regulates the fluid interfaces due to laminar nature.

**Fig. 2:**
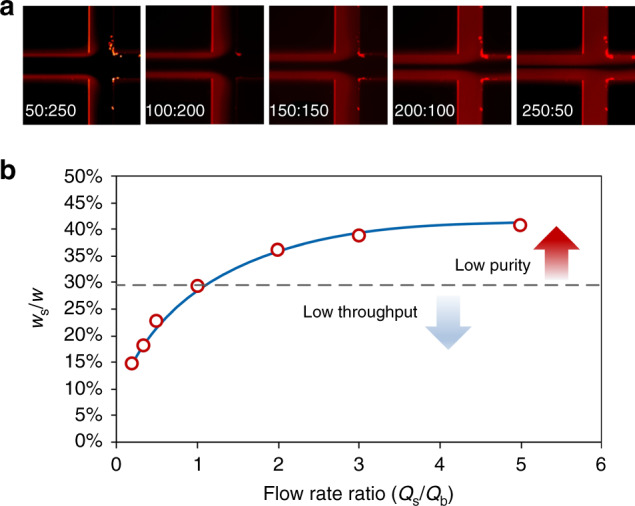
Effect of flow rate ratio on the stream pattern and output sample quality. **a** Fluorescent images showing the pattern of sample and buffer streams depending on input flow rate ratio (*Q*_s_/*Q*_b_) indicated at the lower left corner of each image. Rhodamine B was used to show the otherwise invisible sample flows at sides. **b** Measured width ratio as a function of flow rate ratio. Due to the symmetry, the width ratio was calculated as the average width (*w*_s_) of top and bottom sample flow over channel width (*w*) before the trifurcation. Total flow rate was fixed at 300 µL/min

The two sample–buffer interfaces move either toward or away the channel centerline as a function of flow rate ratio. To identify positions of the interfaces, a solution of Rhodamine B and DI water were first used as sample and buffer flows, respectively (Fig. 2a). The flow rate ratio is defined as the sample flow rate (*Q*_s_) over the buffer flow rate (*Q*_b_). The total flow rate (*Q*_t_ = *Q*_s_ + *Q*_b_) was fixed at 300 µL/min, equivalent to *Re* = 50. Increasing the ratio led to the shift of interfaces toward the channel center. When the ratio reached unity (*Q*_s_:*Q*_b_ = 150 µL/min:150 µL/min), the interface positions were about 29.3% of channel width (*w*) from each sidewall. At this ratio, the two flows appeared to be completely separated by the outlet system. Further increasing the ratio moved the interfaces closer to the centerline and resulted in a portion of sample flow entering the inner outlet (IO; target outlet for CTCs), reducing the purity of target cells. We note here that the width ratio does not linearly respond to flow rate ratio, which is attributed to the uneven velocity profile of channel flow (Fig. 2b).

To ensure separation performance, the flow rate ratio cannot exceed unity for our device. Rather, the ratio has to be set smaller considering the lateral migration of all cells due to inertial effect. All cells initially near the interfaces will migrate into the buffer flow in the middle, leading to compromised purity even when *Q*_s_/*Q*_b_ = 1. Due to the strong size-dependent migration of cells, it is ideal to initially confine all the cells close to sidewalls, so that larger cells will laterally migrate away rapidly and are differentiated from the rest of the cells^32,33^. Thus, the separation resolution and purity can be maximized. However, this would require infinitely small flow rate ratio, which is not preferred since it also means impractically low throughput. An optimal flow rate ratio has to be determined to maximize the throughput while keeps the performance well-tuned.

**Fig. 3:**
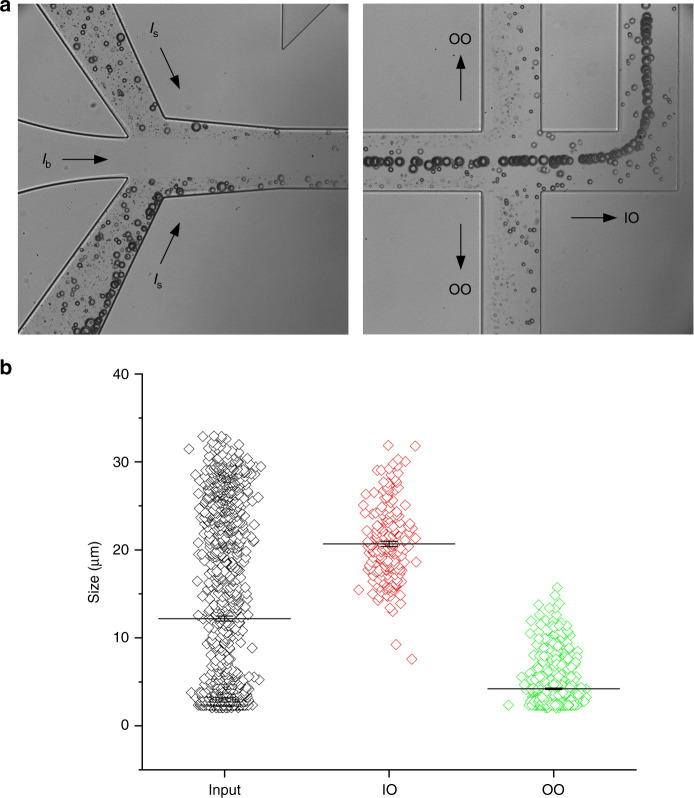
Determination of cutoff size of the microchannel using microparticles with continuous size distribution (2–32 µm). **a** Bright field images showing the size variation of the microparticles, configuration of the flows, and the sorted particle streams at output. **b** Measurements of particle size for input sample and samples collected from inner outlet (IO) and outer outlet (OO). The total flow rate was 300 µL/min and the input flow rate ratio (*Q*_s_:*Q*_b_) was 1:2

Cutoff size is employed to determine this optimal flow rate ratio. A cutoff size is defined as the cell size above which cells are collected from the IO or target outlet. We used polymethyl methacrylate (PMMA) particles which have a continuous size distribution (2–32 µm) to mimic cells in suspensions. As shown in Fig. 3a, particles entered the microchannel with all sizes randomly dispersed and they were ordered such that the larger the particle, the closer it is to the channel center, forming a size gradient of particles along channel width with maximum at the center^33^. When these particle trains pass the trifurcation region, they are separated by the three output-branch channels. Particles larger than a certain critical diameter enter the IO and all the rest flow into the outer outlet (OO). In this case, cutoff size is the critical diameter. As discussed in our early paper^33,38^, the cutoff size can be significantly affected by the resistances of output-branch channels. We set the resistances about equal in this work and thus, let the flow condition to tune the cutoff size.

Cutoff size can be readily obtained from the plot of sizes measured from the three samples (Fig. 3b). At least 200 particles were randomly measured for each sample. The initial size distribution of particles (input) shows a continuous range from 2 to 33 µm. After processing through our channel at a total flow rate of 300 µL/min with a flow rate ratio of 1:2, the distribution was cut into two halves for the two outlets, with >14 µm particles observed in IO and those below 14 µm in OO. Only 2.5% of those particles from IO were below 14 µm in diameter and 0.2% of those from OO were above 14 µm. Hence, the cutoff size is confirmed as 14 µm for our channel at this specific flow condition.

**Fig. 4:**
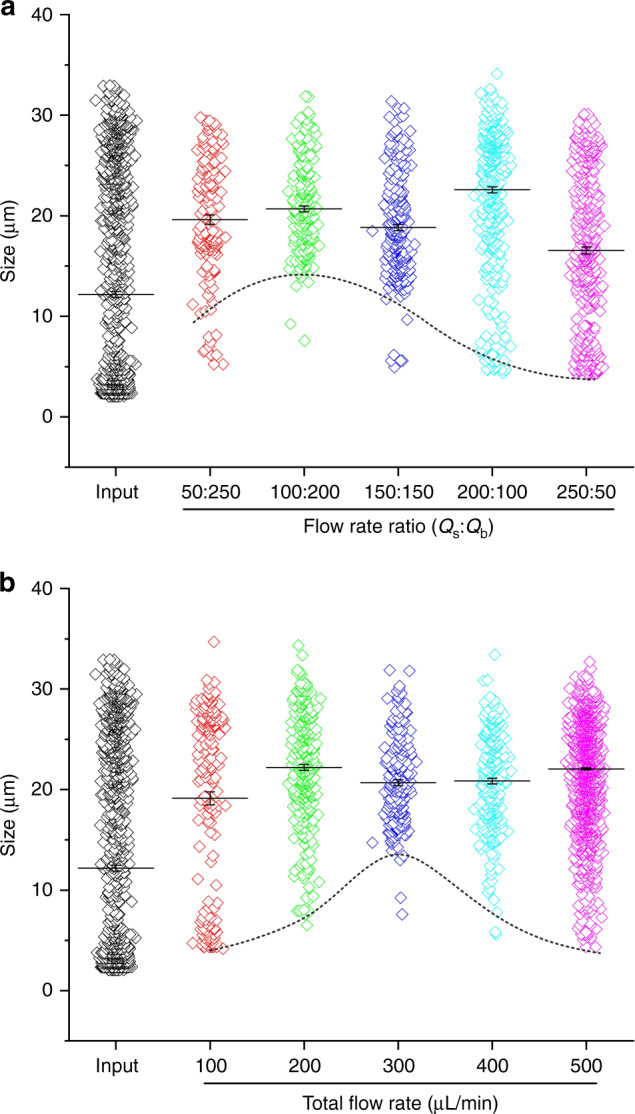
The effects of flow rate ratio and total flow rate on channel cutoff size. **a** Size distributions of samples collected from inner outlet (IO) for five flow rate ratios. Total flow rate was fixed at 300 µL/min. **b** Size distributions of samples collected from IO for five flow rates. Flow rate ratio was fixed at 1:2 in this case

Other flow conditions were subsequently examined to identify the optimal flow condition. The effect of flow rate ratio was first investigated in terms of particle separation performance. Previously, we have discussed how this ratio affects the flow fractionation at the end of the main channel without considering particle and cell motility due to inertial effect and qualitatively analyzed its effect on separation performance (Fig. 2). Here, we ran aforementioned PMMA particles into the device at various flow rate ratio and measured terminal size distributions from each outlet. Figure 4a shows the size distributions of particles collected from IO for five flow rate ratios. The total flow rate was fixed at 300 µm. Since a single species of the particle was used here, the aforementioned parameter “purity” is no longer appropriate in assessing the performance. Cutoff size is used as it inherently reflects “purity” as well. For instance, lower purity means more smaller particles collected in IO and thus the cutoff size shifts downward.

Flow rate ratio alters the cutoff size of our microchannel with fixed layout (Fig. 4a). Increasing the ratio from 1:2 to 1:1 shifts the cutoff size from 14 to 12 µm. This downward trend is consistent with the results and discussion from flow fractionation experiment in Fig. 2. Since the flow rate ratio essentially defines the initial lateral positions of particles (between nearest sidewall and flow interface), increase of this ratio moves interfaces closer to channel centerline where particle focusing positions locate and thus migration distance decreases. Consequently, even smaller particles can reach full focusing and enter the central output-branch toward target outlet for larger particles. This is evidenced by a further decrease of cutoff size to ~4 µm at higher flow rate ratios of 2:1 and 5:1. While we expected a slight upward shift of cutoff size for the ratio below 1:2, our measurements indicate a reduced cutoff size. This unexpected result is most likely due to the fact that more frequent agitation of sample syringe during the course of collecting a sample by shaking the sample syringe to mitigate the influence of particle sedimentation (PMMA particles are heavier than DI water). Since sample flow rate was lower when the ratio decreased, longer time was required and hence more agitation was implemented, which may lead to the inaccuracy of measured cutoff size at lower flow rate ratio when the total flow rate was fixed.

Total flow rate is another key determinant for cutoff size of an inertial microchannel. This is because of the flow rate controls the residential time for particle migration within a microchannel^33^. The initial selection of total flow rate at 300 µL/min is due to the fact that this flow condition (*Re* = 50) requires the shortest channel length for focusing based on our early work^32^. Figure 4b shows the size distributions of samples collected from IO for five total flow rates with an optimal flow rate ratio of 1:2. These results confirm the nonlinear trend that neither higher nor lower total flow rate is preferred. The cutoff size shifts downward for either increasing or decreasing the flow rate, which means deteriorated purity in case of cell separation. Higher flow rate enhances the inertial forces that drive particles toward buffer solution and lower flow rate on the other hand ensures longer residential time for particle migration^32,33^. As a result, 300 µL/min is picked as an optimal total flow rate for all subsequent cell separation experiments.

### Separation from blood

**Fig. 5:**
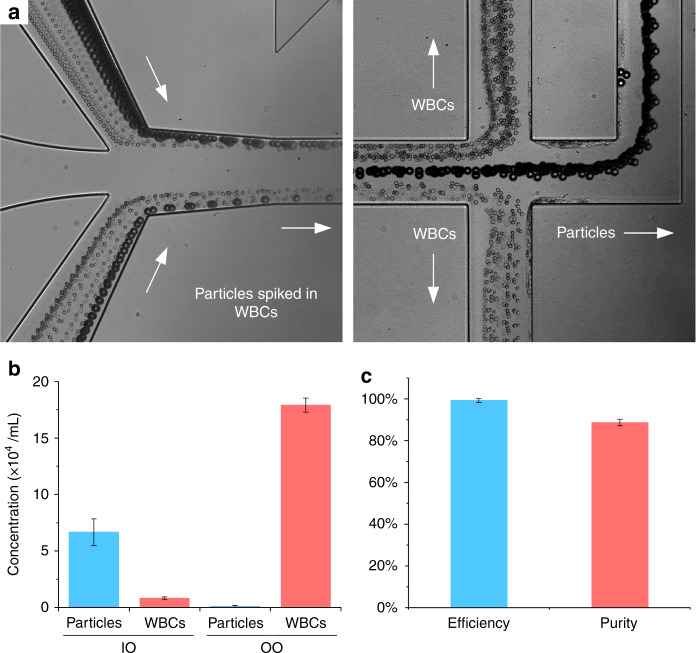
Separation of spiked particles from white blood cells (WBCs). **a** Bright field images of particles and white blood cells at input and output. Particles were sized 18.7 μm in diameter which is larger than most of WBCs and exiting from the central output channel. **b** Concentrations (*N* = 3) of WBCs and particles collected from inner outlet (IO) and outer outlet (OO). **c** Efficiency and purity for particles collected from IO. Total flow rate was 300 μL/min and flow rate ratio was 1:2 between sample and buffer

Before running patient samples, we used WBC suspension spiked with microparticles to further validate and quantify the performance of our multi-flow microchannel. WBCs were isolated from human whole blood by lysis of RBCs. These WBCs were diluted five times in phosphate buffered solution (PBS) and spiked with polystyrene particles (18.7 µm in diameter, CV = 4%). These particles were chosen to mimic the behavior of CTCs which are commonly found to be larger than blood cells for NSCLC^39^ and as we confirmed in the next section. PBS instead of DI water was used as a buffer solution running into the microchannel. Total flow rate was 300 µL/min and sample flow rate was 100 µL/min. Bright field images of input and output (Fig. 5a) suggest the separation was readily achieved. The larger uniformly-sized particles were observed to migrate away from WBC flow near sidewalls and focused into the channel centerline before reaching trifurcation region. The gaps between the focused particle stream and WBC suspension are so evident that the separation performance can be high.

Concentrations measured from samples re-affirm the high-profile performance of our multi-flow microchannel. Almost all particles were present in the sample of IO which is our target outlet. The majority of WBCs (~180,000) were found in sample from OO, with less than 10,000 WBCs (~5.2% of total WBCs) found in the target sample (Fig. 5b). Considering there are normally about 5% WBCs larger than 15 µm, these results are rather encouraging. The performance was quantified in terms of typical parameters, efficiency, and purity (Fig. 5c). Separation efficiency is defined as the ratio of target cells (particles in this case) from IO over the total number of cells collected from both outlets. This efficiency was above 99.3% as suggested by the concentration measurements. The purity of particles was 88.7%. Purity is the ratio of number of target cells (here particles) over total cells (here particles and WBCs) present in the sample of target outlet (IO). Our purity is much higher than the cascaded spiral device^40^ and is comparable to the vortex-based channels^39,41,42^, while our separation efficiency is superior (see Table 1). Considering the large population of WBCs in whole blood, further improvement of purity would be beneficial, and different metrics such as WBCs/mL which were used to quantify the performance of slanted spiral channel^26^ can be preferred, despite a more accurate counting method is required.

**Table 1:** Performance comparison of some inertial-based microfluidic devices for separation of circulating tumor cells

Channel	Blood sample	Dilution	Efficiency	Recovery rate	Purity	Mini spiking (cells/mL)	Flow rate (mL/h)	Comment	Ref.
Straight	Whole blood	No dilution	>89%	~82%	N/A	N/A	6.75	1 cm straight channel	31
Straight	Whole blood	No dilution	95%	77.5%	N/A	50	0.45	Long channel, PEO contamination	30
Spiral	RBC lysed	5×	N/A	74.5%	N/A	72	5	Double spiral + filtration membrane	29
Spiral	RBC lysed	2×	N/A	>80%	400–680 WBCs/mL	66	51	Large slanted spiral, high throughput	26
Spiral	RBC lysed	50×	N/A	>73%	63%	>1000	2.4	Cascaded spirals + zigzag channel	44
Vortex	Whole blood	10–20×	<27%	>57%	89%	200	22.5	8 Paralleled channels, non-continuous	39,48
Straight	RBC lysed	5×	99.3%	>93%	88.7%	2	1.2	Single channel, recovery rate was 83% for spiking 10 cells in 5 mL sample	This work

All flow rates from references are converted into effective flow rate of # mL of undiluted whole blood per hour (mL/h) in the table

**Fig. 6:**
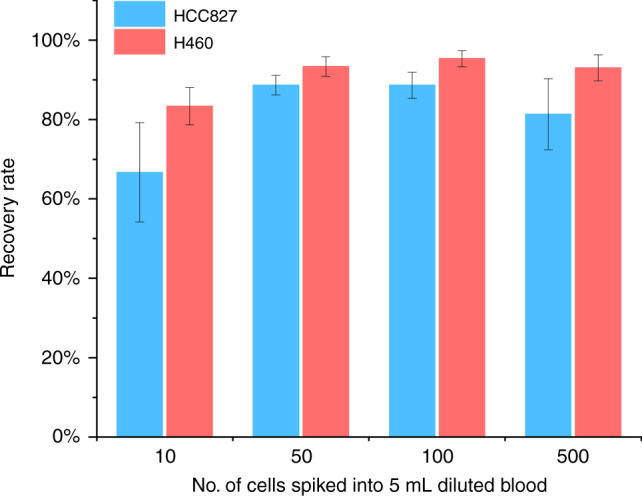
Recovery rate of spiked cells from blood. The recovery rate was determined by spiking cells of two cell lines HCC827 (ATCC^®^CRL2868™) and H460 (ATCC^®^CRL177™) into 5 mL of diluted blood preparations

Performance of our microchannel remains outstanding in isolation of rare cells. This reliability has been demonstrated using cells of two lung cancer cell lines. Cancer cells were spiked into WBCs suspension at physiologically relevant concentrations. Since the concentration of CTCs can be below 1 cell per mL, we spiked WBCs suspensions with 10, 50, 100, and 500 cancer cells per 5 mL of diluted WBC suspension. These cells were recovered after processing through our microchannel and detected by cytocentrifugation onto glass slides and phenotyping. The results are reported in Fig. 6 in terms of recovery rate which is the ratio of recovered cell number over total number of cells injected. In general, the recovery rates for the two cell lines (H460 and HCC827) are very promising as compared to other methods. For cell line H460, the recovery rate is more than 93% for spiked cell numbers above 50. Such recovery rate is better than similar devices reported by others (Table 1). Even for low spiking concentration (10 cells), the recovery rate remains high (>83%) which is even better than the affinity-based HB-Chip^43^. For the other cell line, the recovery rate decreases slightly, but it can still reach 80% or more. These results show much higher sensitivity of our device as compared to others^16,39,40,44^.

### Isolation of CTCs from patient samples

**Fig. 7:**
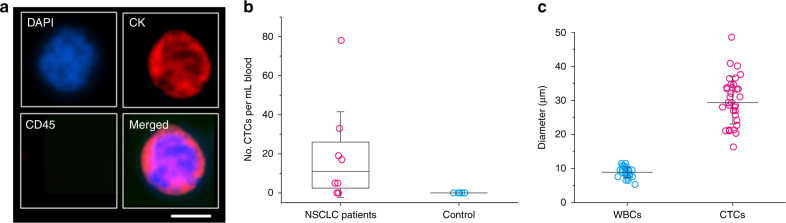
Detection of CTCs in non-small-cell-lung-cancer (NSCLC) patients. **a** Fluorescent images showing detected CTCs from patient samples. Cells were stained by DAPI, CK, and CD45. CTCs are DAPI^+^, CK^+^, and CD45^−^. Scale bar represents 10 μm. **b** CTCs detected from NSCLC patients and healthy donors (control). Results were from 2 mL whole blood. **c** Measurements of cell diameters of circulating tumor cells (CTCs) and WBCs

CTCs were successfully isolated from patient peripheral blood using our multi-flow microchannel. First, 2 mL patient blood (lung cancer stage IV) were incubated with lysis buffer to remove RBCs and all nucleated cells were resuspended in 10 mL PBS (equivalent to 5× dilution of original whole blood). Samples were then run through our microchannel and outputs of the IO were collected and analyzed. The collected cells were stained by DAPI, CD45, and pan-CK immunologically (CellSearch antibody cocktail, Menarini Silicon Biosystems, Italy). CTCs are defined as a DAPI^+^, CD45^−^, CK^+^ cell with high nucleus to cytoplasmic ratio and morphologically larger than background leukocytes. On the other hand, WBCs are DAPI^+^, CD45^+^, and CK^−^. Figure 7a shows a detected CTC in fluorescent field, which is 17 μm in size and exhibits very high nucleus to cytoplasmic ratio. CTCs were detected in 6 of the 8 patients, with a median of 12 CTC per mL and a maximum concentration of 78 CTCs per mL whole blood (Fig. 7b, c). No CTCs were detected in the healthy controls (*N* = 5).

The sizes of detected CTCs were larger than WBCs in general (Fig. 7d). The average size of WBCs measured was 9 µm. On the contrary, the minimum size of isolated CTC was 16 µm and the average size was 30 µm for all the detected CTCs, which is consistent with measurements by others^39^. These results confirm our early estimation of CTC size. While there might be undetected CTCs below the cutoff size of our device, we expect that most of CTCs in the patient sample can be isolated through our device considering the high recovery rate even at low concentration (see Fig. 6). The clear difference between WBC and CTC sizes ensures the high performance of our device in label-free detection of CTCs from NSCLC patient samples.

## Conclusions

In this work, we demonstrated a novel MFM system for label-free separation of CTCs from patient peripheral blood. We systematically investigated the influence of flow rate ratio and total flow rate on the separation performance in terms of cutoff size. Our results using spiked samples show >99% efficiency and >87% purity which is not achieved in similar inertial devices. Our system is suitable for reliable isolation of rare cells such as CTCs as it offers >83% efficiency even for target cell concentration below 10 cells per 5 mL. This reliability has also been validated by detection of CTCs from NSCLC patient blood. The device could be extended to the isolation of CTCs of other cancers and for separation of other sources of liquid biopsy, as long as the targets are larger than blood cells. As demonstrated in this work, the cutoff size of our device is predictable and tunable by accounting for the migration mechanism reported in our early work^31,32^. Tumors of large cells, such as breast and liver cancers^31,45^, can also be detected via liquid biopsy of CTCs in our device. We envision that our system could be a promising alternative to existing technologies toward the unmet clinical need for cancer liquid biopsy and ultimately personalized medicine.

One of the limitations using our novel MFM device for CTC/CTM isolation is that performance in terms of sensitivity and recovery will be compromised when significant size overlap of target and non-target cells exists. CTCs have a wide distribution of size depending on cancer type^45^. While CTCs are generally thought to be larger^46^, some CTCs are even smaller than blood cells^45^. This wide spectrum of cell size may explain that no CTC was detected from 2 of the 8 samples, or that these 2 patients did not have CTCs at that time point (Fig. 7b). Nevertheless, this unexpected result is most likely due to the small sample volume available in this work. We searched for CTCs in only 2 mL whole blood instead of standard 7.5 mL blood. Considering the interpatient heterogeneity of CTC population (Fig. 7c), the 2 mL blood tested might not contain CTCs. Consequently, appropriate sample volume is required to obtain a clinically relevant outcome. Another limitation also stems from the principle of size-based separation. While our approach is independent of the expression of tumor-associated markers, downstream immunofluorescence and molecular characterization are necessary to advance the clinical applications of liquid biopsy.

Another limitation of the current work as compared to similar label-free devices (Table 1) is the relatively low flow rate. Although our device shows much better performance in terms of efficiency, recovery rate, and sensitivity, the effective throughput is 1.2 mL of whole blood per hour which necessitates further improvement. Parallelization is an easy and practical way to achieve higher throughput and it has been employed widely to boost the throughput for spiral^47^ and vortex^48^ channels. In fact, our multiple flow configuration has the potential to process untreated whole blood as demonstrated in our recent work^31^ showing minimal interference during CTC separation from hepatocarcinoma patients. As a result, the throughput can dramatically increase to 6.75 mL whole blood per hour in a single channel. This would lead to one-step isolation protocol that does not require repeated enrichment, which not only reduces labor but also minimizes the potential risk of cell loss.

A potentially exciting application of our technology is in the separation of circulating tumor microemboli (CTM). CTMs are large aggregates of heterotypic cells in the bloodstream^49^ and are more aggressive than single CTCs. Considering the stronger inertial migration for larger target, isolation of CTMs can be readily accomplished in our system. One caveat when applies our device in CTM (or CTC cluster) separation is that our channel height (50 µm) might be smaller than diameters of CTMs, which may pose clogging issue. Nevertheless, this is not necessarily true considering the recent work^50^ which showed high flexibility and plasticity of CTMs/CTC clusters, permitting their transport through extremely small capillary vessel. Neither did we observe clusters from NSCLC patients nor experienced clogging during the experiments. Hence, our MFM device is flexible and versatile in nature, with potential for detection of CTCs/CTMs of various cancers.

## Methods and materials

### Fabrication

Microchannels were fabricated via standard soft photolithography. We used dry film resist instead of the conventional SU-8 photoresist, which simplifies the fabrication process and provides superior uniformity in terms of channel height. Briefly, dry film resist (ADEX 50, DJ MicroLaminates Inc., Boston, MA, USA) was used to pattern microchannels on a 3″ silicon wafer by photolithography (I-line 365 nm). Polydimethylsiloxane (PDMS, Sylgard 184, Dow Corning, Midland, MI, USA) was cast on the wafer and peeled after 6-h cure on a 65 °C hotplate. Replicated straight channels (150 μm wide, 50 μm high, and 20 mm long) in PDMS were bonded to 1″ × 3″ glass slides (Fisher Scientific Inc., Hampton, NH, USA) using surface plasma treatment (PE-50, Plasma Etch Inc., Carson City, NV, USA). The inlet and outlet ports were punched manually using stainless flat head needles before the channels were sealed onto glass slides.

### Microparticle suspensions

Non-cellular samples were first prepared to characterize the microchannel. Rhodamine B was used to identify fluid interfaces between sample streams and buffer stream in the microchannel. Microparticles (Cospheric LLC, Santa Barbara, CA, USA) with continuous size distribution (2–32 µm) were used to characterize the cuff-off size of the microchannel. These particles are made of PMMA and have a density of ~1.2 g/cc. Particle powders were suspended in DI water (132 mg powder in 50 mL DI water), plus a drop of Tween-80 (Fisher Scientific Inc., Hampton, NH, USA) preventing particle aggregation. Microparticles with a diameter of 18.7 µm (CV 4%) from PolySciences Inc. (Warrington, PA, USA) were used to mimic the behavior of large CTCs. The volume fraction of these particles was 0.05% in WBC suspensions.

### WBC separation

Prior to test patient samples, we used WBC suspensions spiked with 18.7 µm particles to quantify the performance of our microchannel. WBCs were isolated from human whole blood (Zenbio Inc., Research Triangle Park, USA) via lysis of RBCs and centrifugation. Briefly, 1 mL blood was added into 15 mL ACK lysis buffer (Fisher Scientific Inc., Hampton, USA) and was incubated on a shaker at room temperature for 5 min. The sample was centrifuged at 300*g* for 5 min at 4 °C. After the removal of the supernatant, the white cell pellet was resuspended in 5 mL PBS and re-centrifuged at 300*g* for 5 min. The cell pellet was re-suspended in 5 mL PBS (equivalent to 5× dilution of WBCs of original whole blood). The 18.7 µm particles were subsequently spiked into the WBC suspension with volume fraction of 0.05% for characterizing channel performance.

### Imaging and data analysis

Both regular camera and high-speed camera were used to image flows inside our microchannel. Andor Zyla 5.5 camera (Andor Technology Ltd., Belfast, UK) was used to image the flow configuration within the microchannel in fluorescent field. Rhodamine B dye running through sample inlet was imaged using mCherry filter cube and the obtained images were later pseudo-colored red for visualizing the fluid interfaces between sample and buffer flows. The widths of Rhodamine B streams were measured from the intensity profile using ImageJ^®^ (NIH, USA). A high-speed camera (FASTCAM Mini AX 200, Photron USA Inc.) was used to capture particles and cells flowing inside our channel. Exposure was set to 1 µs to capture individual particles and cells traveling at high speed in the microchannel. Thousands of frames were taken for later analysis using ImageJ^®^.

### Sizing and counting

Samples collected from each outlet were analyzed to acquire the sizing and concentration information. Briefly, samples were shaken using Vortex mixer (Thermo Fisher Scientific, Waltham, MA, USA) for at least 30 s to ensure the random disperse of particles. A drop of sample was applied onto a clean glass slide and bright field images were taken after particles were settled on the slide. Images were analyzed using “Analyze Particle” module of ImageJ® to gather the sizing information. At least 200 particles were measured for each sample to improve the accuracy. Particle and cell counting were carried out in disposable hemocytometry (INCYTO C-Chip™, Fisher Scientific Inc., Hampton, NH, USA). Three tests were implemented to get sample concentration. The efficiency was calculated as the ratio of particles collected from target outlet (IO) over total number of particles collected from both outlets. The purity was the percentage of particles in target outlet (number of particles over total number of both particles and WBCs).

### Chip characterization using cell lines

Before patient samples were tested, the performance of the straight microfluidic chip was assessed using NSCLC cell lines HCC827 (ATCC^®^CRL2868™) and H460 (ATCC^®^CRL177™) which were a generous gift from A/Prof Derek Richards (QUT, Brisbane). Cells were cultured under standard conditions in humidified incubators at 37 °C, 5% CO_2_ in RPMI1640-Glutamax (Life Technologies Inc., Carlsbad, CA, USA) supplemented with 10% fetal bovine serum (FBS) and 1% penicillin/streptomycin. Cells were lifted using TrypLE Express reagent (Thermo Fisher Scientific, Waltham, MA, USA), resuspended in media and gently mixed on a shaker prior to experiments. Cell line authenticity was confirmed by short tandem repeat (STR) profiling with Stem Elite™ ID system (Promega Corp., Madison, WI, USA) according to manufacturer’s instructions. Cell lines were confirmed to be negative for mycoplasma infection by Hoechst staining and PCR. Efficiency tests were conducted by performing dilutions of the cell suspensions in 10× diluted healthy blood. The following clinically relevant cell numbers were used to inform on enrichment efficiencies: 500, 100, 50, and 10 cell spike-in experiments. The cells were collected and stained for pan-cytokeratin, CD45, and DAPI. The percentage recovery was calculated as the number of cancer cells captured through the CTC outlet of the chip over the total number of cancer cells spiked into the sample.

### Patient recruitment

This study was conducted at the Princess Alexandra Hospital in Brisbane, Queensland, Australia. Ethics approval was obtained from the Metro South Health District Human Research Ethics Committee in accordance with the National Health and Medical Research Councils guidelines: Approval number HREC/11/QPAH/331. This study also has institutional approval from the Queensland University of Technology Human Ethics Committee: Approval number 1100000420. Following written informed consent, 8 mL of blood was collected in K2E EDTA vacutainers from a total of 13 participants (*n* = 8 NSCLC patients and *n* = 5 healthy volunteers). All NSCLC patients were treatment-naive at the time of blood collection.

### Enrichment and phenotyping of CTCs

Blood samples collected from the participants were lysed using RBC lysis buffer (Astral Scientific, Australia) and centrifuged at 500×*g* for 15 min. The cell pellet was resuspended in 10 mL of 1× PBS solution. The pre-diluted sample was loaded onto a 10 mL Terumo syringe and passed through the inlet of the chip at 0.1 mL/min whilst the sheath buffer (1× PBS, 2 mM EDTA and 0.5% BSA) was pumped through at 0.2 mL/min. The CTC sample outlet was collected in a sterile 15 mL falcon tubes (Corning Inc., Corning, NY, USA) and centrifuged at 300×*g* for 5 min to obtain a pellet. The cell pellet was resuspended in 4% paraformaldehyde and cytocentrifuged (Cytospin™ 4, Thermo Fisher Scientific, Waltham, MA, USA) at 800 rpm for 5 min onto glass slides for phenotyping. Cells were permeabilized in 0.2% (v/v) Triton X-100 for 10 min at room temperature, followed by blocking with 10% FBS (Invitrogen) for 1 h at room temperature. Slides were incubated with the CellSearch antibody cocktail including DAPI (Menarini Silicon Biosystems, Italy) overnight at 4 °C. Slides were washed 3 times in PBS, allowed to air dry, and mounted with antifade prolong gold (Thermo Fisher Scientific, Waltham, MA, USA). The cytospots were coverslipped and imaged on the Zeis Axio Imager 2 (Oberkochen, Germany). CTCs were visually inspected and classified as CTCs after meeting the following criteria: (1) cytokeratin-8, 18, 19 positive (2) CD45 negative (3) high nucleus to cytoplasmic ratio (4) morphologically larger than background leukocytes (5) intact nuclei. CTC clusters were reported as CTCs in close proximity and CTM when CTC clusters included leukocytes (CD45 positive cells). The results were reported as the number of CTCs per 2 mL whole blood.

### Cell imaging

Scanning for CTCs was performed on the Zeiss Axio Z2 microscope (Oberkochen, Germany) and sequential images were captured after fluorescent staining. A multi-exposure protocol was used to detect the signals. The Zen software was used to interrogate the images and constrained iterative algorithms used for image deconvolution.
